# Tumor antigen-associated autoantibodies: generation mechanisms, roles in tumorigenesis and progression, and clinical application prospects

**DOI:** 10.3389/fimmu.2026.1717454

**Published:** 2026-05-14

**Authors:** Wenqi Tai, Cuiling Zheng

**Affiliations:** Department of Clinical Laboratory, Laboratory Department, National Cancer Center/National Clinical Research Center for Cancer/Cancer Hospital, Chinese Academy of Medical Sciences and Peking Union Medical College, Beijing, China

**Keywords:** early diagnosis, mechanism of generation, prognosis evaluation, tumor antigen-associated autoantibodies, tumor promotion, tumor suppression

## Abstract

Tumor antigen-associated autoantibodies (TAAbs) are generated by the immune system in response to tumor-associated antigens (TAAs). The underlying mechanisms of their production remain incompletely understood, yet may encompass factors such as aberrant protein expression, deficiencies in immune tolerance, the tumor inflammatory microenvironment, exosomes, and immunogenic cell death. TAAbs exert a dual effect in tumor initiation and progression, both facilitating and impeding the development of malignant tumors. They represent promising biomarkers for early tumor diagnosis, prognosis assessment, and evaluation of treatment efficacy or outcomes, thereby emerging as a current research focal point. This review initially clarifies the multidimensional mechanisms and dual functions of TAAbs in tumorigenesis and tumor progression, and subsequently explores their application prospects in tumor diagnosis, treatment, and prognosis. Finally, through a summary and analysis of the current research landscape, three crucial and feasible future research directions are put forward. It should be underscored that TAAb detection is a promising adjunctive instrument for tumor diagnosis and treatment; however, it still confronts substantial challenges in clinical application and necessitates further in-depth exploration.

## 1 Introduction

Tumor continues to be a significant global public health concern, characterized by consistently elevated incidence and fatality rates. Thus, its diagnosis, treatment, and prognosis remain significant issues need immediate medical intervention.Tumor-associated antigens (TAAs) are a distinct group of cellular proteins found in tumor patient serum that trigger spontaneous autoantibody reactions. In the diagnosis and treatment of tumor patients, serum TAAs are one of the most often tested biomarkers. Conventional serum levels of TAAs, which are frequently used to track therapy effectiveness and evaluate prognosis, are challenging to identify in the early stages of tumor since they rise with tumor size.

Tumors develop when the immune system’s surveillance role fails, permitting tumor antigens to evade detection, elimination, or immune evasion in multiple ways. The creation and exposure to TAAs provoke the activation and proliferation of B lymphocytes, resulting in the production of tumor antigen-associated autoantibodies (TAAbs). Concurrently, they can be identified, seized, processed, and displayed to T lymphocytes by antigen-presenting cells, including macrophages and dendritic cells. This step stimulates the secretion of cytokines and chemokines, establishing a cascade that enhances autoantibody production.TAAbs are remarkably persistent proteins that can be detected months to years prior to the onset of clinical signs in tumor patients, remaining present long after tumor antigens have vanished (1, 2). This characteristic establishes TAAbs as prospective biomarkers for early tumor diagnosis, prognostic evaluation, and therapy efficacy assessment. This review seeks to systematically elucidate the mechanisms of TAAbs synthesis, their intricate roles in tumor progression, and their potential clinical applications. We examine existing constraints in TAAbs research and prospective avenues for future studies, aiming to offer novel insights for tumor immunodiagnosis and immunotherapy.

## 2 Mechanisms of TAAbs generation

The production of TAAbs offers direct evidence of active immune responses during carcinogenesis; however their underlying mechanisms are not fully comprehended. According to the current literature, the principal mechanisms can be delineated as follows:

### 2.1 Protein overexpression, aberrant expression, and structural modifications

#### 2.1.1 Overexpression of normal proteins

In breast tumor, the human epidermal growth factor receptor 2 (HER2) is overexpressed. The immune system identifies the excessive amount of wild-type protein, triggering the body’s immunological response and resulting in increased concentrations of anti-HER2 autoantibodies in the bloodstream (3).

#### 2.1.2 Spatiotemporal aberrant expression of normal proteins

In normal hepatocytes, centromeric protein F (CENPF) is predominantly expressed exclusively within the nucleus of proliferating cells. But in hepatocellular carcinoma, it is overexpressed in the cytoplasm and nuclei of all cells. In a murine hepatocellular carcinoma model, CENPF overexpression is significantly correlated with tumor proliferation and the synthesis of its autoantibodies (4). Cancer-testicular antigens (CTAs) are typically expressed in immune-privileged locations, like the testes, ovaries, or trophoblastic cells of the placenta (5). In tumor patients, they can be aberrantly produced in tumors of various tissue sources, resulting in the generation of matching autoantibodies (6), as observed in non-small cell lung tumor (7).

#### 2.1.3 Protein structural alterations

Modifications in protein structure can reveal concealed antigens. Mucin 1 (MUC1), located on the surface of epithelial cells in the respiratory, urogenital, and digestive tracts, has its core peptide usually obscured by peripheral glycan chains and is generally expressed at low levels. In tumor cells, elevated glycosyltransferase activity results in inadequate glycosylation, producing shorter side chains that expose the previously hidden core peptide, thereby triggering the generation of autoantibodies (8).

All tumors contain somatic mutations. Using tumor protein 53(p53) mutations as an illustration, the mutant p53 protein exhibits a loss of activity and an increase in stability, inhibiting the hydrolysis of substantial quantities of structurally aberrant proteins. These substances accumulate in the body and provoke the synthesis of autoantibodies (9, 10). Moreover, atypical post-translational modifications of proteins—such as glycosylation, methylation, phosphorylation, ubiquitination, acetylation, citrullination, and adenosine diphosphate-ribosylation—can elicit immune responses (11), potentially creating novel antigenic epitopes or augmenting the presentation of self-epitopes, thus increasing affinity for the major histocompatibility complex (MHC) or T cell receptors (12).

### 2.2 Deficiencies in immune tolerance

Tumor-infiltrating B cells are present in the majority of malignancies (13), and tumor-associated tertiary lymphoid structures promote B cell activation, proliferation, and differentiation (14), indicating a violation of local self-immunity tolerance. Comprehensive studies demonstrate that the presence of B cells or plasma cells within tumor tissues is associated with increased survival rates in patients with multiple tumors, including melanoma, breast tumor, colorectal tumor, hepatocellular carcinoma, and pancreatic tumor (15–19). These B cells can generate specific autoantibodies that identify antigens present on the surface of or secreted by tumor cells, therefore, modulating tumor cell proliferation. Specific autoantibodies can directly impede tumor progression, avert recurrence, or metastasis by activating T lymphocytes, stimulating dendritic cells, or disrupting connections between receptors on the surface of tumor cells and ligands (20, 21).

### 2.3 Inflammatory microenvironment

Inflammatory cells and immunoregulatory substances inside the tumor microenvironment affect tumor growth and metastasis (22). The enduring inflammatory milieu increases vascular permeability, thereby exposing significant amounts of TAAs to the immune system and prompting the generation of considerable autoantibodies against TAAs in tumor patients (23). Simultaneously, the inflammatory tumor microenvironment emits significant reactive oxygen species and reactive nitrogen species, which inflict damage on cellular DNA and deactivate DNA repair enzymes, resulting in gene mutations that subsequently enhance the synthesis of corresponding autoantibodies (24).

### 2.4 Exosomes and immunogenic cell death

Exosomes released by tumor cells into the microenvironment, including tumor antigens, are identified as foreign by the immune system, eliciting humoral and cellular immune responses (25, 26). Exosomes secreted by pancreatic tumor cells are abundant in tumor antigens, including annexin A1(ANXA1), histone H2A(H2AF), pyruvate kinase M2 Isozyme(PKM2), and heat shock protein A8(HSPA8), which consequently provoke the generation of specific autoantibodies (27).

In the course of tumor therapy, persistent immunological assaults, pharmacological influences, and genomic instability result in the annihilation and demise of tumor cells. The release of tumor cell contents reveals tumor antigens to the immune system, stimulating immunological responses and producing autoantibodies. For example, after combined treatment with intracranial stereotactic radiosurgery and ipilimumab in melanoma patients, autoantibodies against melanoma antigen A3 (MAGEA3) and PAS domain-containing protein 1 (PASD1) were detectable in the body (28).

### 2.5 Tumor-derived “pseudo-protein” molecules (Ig-CA)

Ig-CA refers to non-classical immunoglobulins abnormally expressed by tumor cells. Although Ig-CA shares a similar basic structure with B-cell-derived immunoglobulins, their gene rearrangements are different, and they often display abnormal glycosylation patterns and restricted variable region sequences. Ig-CA is not only present in the cytoplasm and on the cell membranes of tumor cells but can also be secreted into the extracellular space. It can directly promote malignant behaviors such as tumor cell proliferation, migration, invasion, and resistance to apoptosis; induce inflammation; mediate immune evasion; activate platelets; and participate in tumor-associated thrombosis. Therefore, Ig-CA primarily functions as a carcinogenic promoter rather than a product of adaptive immune responses. Many tumors, particularly malignant tumors of epithelial origin (such as cervical tumor, breast tumor, lung tumor, liver tumor, gastric tumor, colorectal tumor, prostate tumor, esophageal tumor, pancreatic tumor, bladder tumor, and nasopharyngeal tumor) and soft tissue tumors (such as malignant fibrous histiocytoma and leiomyosarcoma), as well as certain hematological malignancies such as acute myeloid leukemia, commonly show high expression of Ig-CA (29).

## 3 Role of TAAbs in tumorigenesis and progression

The function of TAAbs in tumorigenesis, progression, and prognosis prediction differs, influenced by both the synergistic or inhibitory interactions between autoantibodies and antigens, as well as the tumor microenvironment.

### 3.1 Antitumor effects

#### 3.1.1 Neutralizing signaling pathways

Elevated levels of TAAbs can attach to TAAs on the surfaces of tumor cells, effectively obstructing the pro-tumor signaling pathways they facilitate. Anti-HER2 autoantibodies inhibit HER2 gene phosphorylation and the downstream phosphoinositide 3-kinase/protein kinase B(PI3K/PKB) signaling pathway, producing anti-tumor effects akin to trastuzumab and enhancing the prognosis of HER2-overexpressing breast tumor (3, 30). MUC1, a transmembrane mucin that is overexpressed, facilitates the transcription of programmed cell death ligand 1(PD-L1) through myelocytomatosis(MYC) and nuclear factor Kappa B p65 subunit(NF-κBp65), thereby promoting immune evasion in tumors and increasing the invasiveness of tumor cells (31). Autoantibodies that target MUC1 with abnormal O-glycopeptide epitopes bind to MUC1, obstructing its immune evasion pathway and consequently inhibiting the progression of breast tumor (32). Alveolar rhabdomyosarcoma exhibiting elevated expression of fibroblast growth factor 8 (FGF8) enhances malignant biological behavior via FGF8-mediated angiogenesis and the promotion of metastasis. Elevated serum anti-FGF8 autoantibody levels inhibit FGF8 downstream signaling pathways, which in turn suppresses malignant biological behavior and enhances patient survival rates (33).

#### 3.1.2 Activation of effector immune cells

TAAbs interact with Fcγ receptors on natural killer cells and macrophages through their Fc segments, facilitating antibody-dependent cellular cytotoxicity (ADCC). Simultaneously, they initiate the complement system to trigger complement-dependent cytotoxicity (CDC). Autoantibody-antigen complexes can be phagocytosed by dendritic cells and subsequently cross-presented to CD8+ T cells, thereby stimulating cellular immune responses that target tumor cells and inhibit tumor progression (27, 30).

### 3.2 Tumor promotion

#### 3.2.1 Enhancement of malignant biological behavior in tumor cells

In pancreatic tumor, autoantibodies targeting annexin A2 (ANXA2), Ezrin(EZR), MutS homolog 2(MSH2), phosphoglycerate kinase 1 (PGK1), and focal adhesion protein (VCL) are linked to the negative regulation of apoptosis and the promotion of cell migration, among other malignant behaviors (34).

#### 3.2.2 Induction of immunosuppression

The ongoing production of autoantibodies and chronic inflammatory responses can result in the infiltration and activation of immunosuppressive cells, such as myeloid-derived suppressor cells (MDSCs) and regulatory T cells (Tregs). This process establishes an immunosuppressive microenvironment that promotes tumor immune evasion (35).

In conclusion, both TAAs and TAAbs produced against them are of significant importance in regulating cell growth and proliferation or exerting an impact on the tumor microenvironment. They engage in diverse mechanisms, such as endogenous apoptosis signaling, cellular processing, catabolic processes, growth regulation, glucocorticoid receptor signaling, the hypoxic response, and alterations in cellular structure, to elicit either antitumor or pro - tumor effects.

## 4 Role of TAAbs in tumor diagnosis and treatment

### 4.1 Biomarkers for early tumor detection

Tumors in various systems can stimulate the generation of autoantibodies (36–40). The diversity in types and quantities of these autoantibodies underpins their utility in early tumor diagnosis. Studies have shown that changes in TAAbs can be detected in peripheral blood up to five years before the onset of clinical symptoms (41, 42). For example, TAAbs can be detected as early as 7.2 years (median 3.5 years) before the radiological detection of lung tumor or up to 8.4 years (median 4.2 years) before the onset of clinical symptoms (43–49). Similar findings have been reported in liver tumor (50, 51), ovarian tumor (52), colorectal tumor (53), and esophageal tumor (54). Furthermore,TAAbs remain stable and consistently present in the bloodstream. Additionally, the immune system’s distinct signal amplification capability allows serum levels of autoantibodies to significantly surpass antigen levels (55). TAAbs exhibit greater efficacy than tumor antigens in serum as biomarkers for the early diagnosis of tumors. The simultaneous detection of tumor-associated antigen autoantibodies is presently a focal point of research. Furthermore, autoantibodies may be integrated with additional biological markers, including TAAs and microRNAs, for concurrent detection.

The utilization of TAAbs for the early diagnosis of lung tumor has attained a considerable level of maturity. The EarlyCDT-Lung autoantibody panel, associated with lung tumor, has been clinically promoted in Europe and the United States (56). Seven autoantibody detection kits developed in China have received approval from the China Food and Drug Administration (CFDA) and are currently utilized for clinical testing in select hospitals (57). Ongoing investigations persist. Jiang et al. (58) reported that the combined detection of anti-Gremlin 1(GREM1), anti-high-mobility group box 3(HMGB3), and anti-PC4 and SFRS1 interacting protein 1(PSIP1) for early lung tumor diagnosis resulted in an area under the curve (AUC) of 0.833, with a sensitivity of 73.39% and a specificity of 86.55%. The simultaneous detection of anti-GREM1, anti-HMGB3, anti-ZW10 interactor(ZWINT), DNA topoisomerase II alpha(TOP2A), and PSIP1 resulted in an AUC of 0.808, with a sensitivity of 70.16% and a specificity of 87.39%. No statistically significant difference in sensitivity or specificity was found between the two combinations for lung tumor diagnosis.

The Videssa Breast assay for breast tumor is commercially available as well (59). Qiu et al. (60) demonstrated that the combined detection of CCAAT/enhancer binding protein alpha(CEBPA), centrosomal protein 55(CEP55) GATA binding protein 3(GATA3), Harvey rat sarcoma viral oncogene homolog(HRAS), patched 1(PTCH1), and v-ral simian leukemia viral oncogene homolog A(RalA) effectively differentiates breast tumor from benign breast diseases, achieving sensitivities of 71.2% and specificities of 90.2%. The combination exhibited a sensitivity of 72.3% and a specificity of 90.8% for the early detection of stage I–II breast tumor in women under 50 years of age.

In ovarian tumor, the combination of the carbohydrate antigen carbohydrate antigen 125(CA125) with anti-CA125 autoantibodies increases diagnostic sensitivity and specificity to 94.7% and 78.2%, respectively (61). Autoantibodies p53 and p62 are regarded as potential biological markers for the early monitoring of hepatocellular carcinoma progression (62, 63). The combined detection of anti-p53, human cervical cancer gene (HCCR), MYC oncogene (C-myc), and microtubule-associated protein D2 (MDM2) resulted in approximately 72% diagnostic concordance in esophageal squamous cell carcinoma. However, the increase in hepatocellular carcinomaR, C-myc, MDM2, and microRNAs (miR-21, miR-223, and miR-375) resulted in a diagnostic concordance of 81% (64).

### 4.2 Applications in tumor therapy, prognostic evaluation, and efficacy assessment

Research increasingly suggests that TAAs could be viable targets for immunotherapy (65). The mechanism by which patients with tumors produce specific autoantibodies against these antigens provides valuable insights for the development of new therapies for refractory tumors (66). Patients with stage IIIB/IV non-small cell lung tumor have shown clinical benefit from the MUC1-targeted tumor vaccine liposomal BLP25(L-BLP25) (67). Additionally, monoclonal antibodies targeting HER2/neu have resulted in favorable clinical outcomes for HER2-positive breast tumor patients (68).

TAAbs serve as prognostic markers for evaluating tumor progression, recurrence, metastasis, and survival across various tumors, including breast tumor (3, 69), lung tumor (70–75), gastric tumor (76, 77), colorectal tumor (78–80), hepatocellular carcinoma (81), prostate tumor (82, 83), pancreatic tumor (84), bladder tumor (85), esophageal squamous cell carcinoma (86, 87), and melanoma (88, 89). P53 autoantibodies have undergone the most comprehensive investigation among these. Studies show that p53 autoantibodies are linked to improved overall survival in patients with hepatocellular carcinoma (90). Additionally, serum p53 autoantibody levels are significantly related to histological grade, clinical stage, and lymph node metastasis in esophageal carcinoma. These levels can also serve as indicators for assessing treatment efficacy before and after radiotherapy in esophageal carcinoma patients (91). The presence of p53 autoantibodies in the serum of gastric tumor patients is significantly linked to poor prognosis (76). In breast tumor patients, elevated serum levels of B-cell lymphoma 2(Bcl-2) and p53 autoantibodies concurrently indicate a worse prognosis (92). Additionally, p53 autoantibodies can predict median progression-free survival and overall survival following surgical treatment in non-small cell lung tumor patients, with higher autoantibody levels correlating with poorer outcomes (70). Zhou et al. (93) demonstrated that an autoantibody profile including p53, breast cancer type 2 susceptibility protein(BRCA2), HuD, tripartite motif-containing protein 21(TRIM21), and the New York esophageal squamous cell carcinoma 1 (NY-ESO-1) serves as an effective predictor of immunotherapy efficacy and survival in patients with advanced lung tumor.

Immune checkpoint inhibitors (ICIs), including programmed cell death-1/programmed cell death ligand-1 (PD-1/PD-L1) and cytotoxic T-lymphocyte-associated protein 4 (CTLA-4), are prominent areas of investigation in tumor immunotherapy. Existing literature indicates that various autoantibodies are associated with the efficacy and prognosis of PD-1/PD-L1 monoclonal antibody treatment, particularly in non-small cell lung tumor. Numerous studies demonstrate that the presence of tumor-associated autoantibodies for NY-ESO-1, X antigen family, member 1(XAGE1), and p53 is associated with prolonged progression-free survival and increased objective response rates in patients with advanced or metastatic non-small cell lung tumor receiving treatment with nivolumab or pembrolizumab (93, 94). Recent studies on autoantibodies aim to assess the efficacy of CTLA-4 monoclonal antibody treatment and predict prognosis, with a primary emphasis on NY-ESO-1 autoantibody. This autoantibody may act as a significant predictor of treatment response to CTLA-4 monoclonal antibodies in patients with advanced melanoma (95, 96).

In summary, **Table 1** presents the applications of TAAbs as biomarkers in the early diagnosis, prognosis prediction, and assessment or prediction of treatment efficacy for common solid tumors over the past five years, while **Table 2** lists the TAAbs detection combinations that have been most frequently studied in each type of solid tumor during this period. We can observe that current research on the application of TAAbs in tumor diagnosis and screening accounts for a significant proportion, with studies on lung tumor being the most mature. The majority of these studies focus on 7-TAAbs composed of P53, protein gene product 9.5(PGP9.5), sex determining region Y-box protein 2(SOX2), G antigen 7(GAGE7), GAGE family member U4-5(GUB4-5), melanoma antigen family A 1(MAGEA1), and cancer/testis antigen(CAGE). Some studies have also reported the diagnostic efficacy of TAAbs in combination with other tumor markers. Additionally, a current research focus involves incorporating age, gender, or other high-risk factors alongside biomarkers such as TAAs and TAAbs to construct various diagnostic risk models using machine learning algorithms. On the other hand, while current research on TAAbs remains primarily focused on IgG autoantibodies, the application of other immunoglobulin types, such as IgM, and IgG subtypes in tumor diagnosis and treatment is gradually gaining attention.

**Table 1:** Applications of TAAbs in the diagnosis, prognosis, and treatment of common solid tumors.

Tumor category	Early-detection biomarkers	Prognosis-predictive biomarkers	Efficacy assessment/prediction biomarkers
Lung tumor	PRTN3 (97), p53,PGP9.5,SOX2,GAGE7,GBU4-5,MAGEA1,CAGE (98), UBQLN1 (99)	MAX,DHX29,TAPBP (100)	p53,BRCA2,HUD,TRIM21,NY-ESO-1 (93)
Bladder tumor	PPP1CA (101),ENO1,VDAC2,AKR1B1,SDF2L1,PRDX6,PSME1,HSPB1,PHB1 (102),N-OH- AABP (103)	PPP1CA (101),ENO1,VDAC2,SDF2L1,PRDX6,PHB1 (102)	BRCA2,TP53,CTNBB1,BICD2,UACA (104)
Colorectal tumor	CCD83,CEA,MAPKAPK3, RPH 3AL,SEC61b (105), NUP54,c90rf80,RSP28, DLAT,VTI2 (106), RPRD1A (107)	FSCN1,ANXA9,ANXA13,DLAT,NUP54,C9orf80,VTI2,OLR1,RPS28 (106), JMJD6 (108)	CDC25B,COX2 (109)
Breast tumor	p53,MUC1,Her2/Neu,c-MYC,NY- ESO -1,IMP2/p62,survivin,CDKN2A(p16),BRCA1,BRCA2,Cyclin B1 (110)	HER2 (3), Se (111)	MUC1 (112)
Ovarian tumor	CTAG1,IL-8 (113), PDLIM1 (114), MMP14 (115), MAGEB4,sharpin,MAGEA12,MAGEA4,MRFAP1L1,PNMA2, AURKA ,SERPINB1,XAGE3,BAGE4 (116)	MMP14 (115), MAGEA4 (116)	-
Liver tumor	BIRC5 (117), UBE2Z,CNOT3,EID3 (118), BRD2 (119), OLA1 (120), CNN2 (121)	BIRC5 (117), OLA1 (120), CNN2 (121)	-
Pancreatic tumor	p53,Ezrin,CLDN17,KCNN3,SLAMF7,SLC22A11,OR51F2 (122)	ENO1,FUBP1 (123)	-
Esophageal tumor	GNA11 (124), POSTN ,TIMP1 (125), SOX2 (126)	p53 (127), WDR1,CFL1 (128)	-
Gastric tumor	FH (129)	FH (129), ENO1,SSNA (130)	-
Prostate tumor	p16,IMP2,HSP60 (131)	-	-
Cervical tumor	-	-	-

-: No relevant studies have been reported in the past five years.

**Table 2:** TAAbs-panel for common solid tumors.

Tumor category	Autoantibody panels	SN(%)/SP(%)/AUC	Reference
Lung tumor	P53+SOX2+SSX1+HuD+NY- ESO -1+CAGE+MAGE-A4+P62+CK20	40.8/90.5/0.64	(132)
	p53+PGP9.5+SOX2+GAGE7+GUB4-5+MAGEA1+CAGE	23.73/88.79/0.527	(133)
	CKAP2+DEPDC1B+DPP4+PARP11+TCP11L2+TIPARP	76.19/55.74/-	(134)
	CKAP2+DEPDC1B+DPP4+PARP11+TCP11L2+TIPARP+ETHE1+CTAG1A+C1QTNF1+TEX264	80.0/87.0/-	(134)
	P53+PGP9.5+SOX2+GAGE7+GUB4-5+MAGEA1+CAGE	61.5/88.5/0.8062	(135)
	TSHR+ERBB2+survivin+PIK3CA+JAK2	56.63/93.98/0.827	(136)
	PRTN3 IgG+PRTN3 IgM+CEA	58.4/88.2/0.783	(101)
	ELAVL4-IgM+GDA -IgM+GIMAP4-IgM+GIMAP4-IgG+ MGMT -IgM+UCHL1-IgM+DCTPP1-IgM+KCMF1-IgM+UCHL1-IgG+WWP2-IgM	70.5/80.0/-	(137)
	P53+PGP9.5+SOX2+GAGE7+GUB4-5+MAGEA1+CAGE	30.0/84.3/0.61	(102)
	P53+PGP9.5+SOX2+GAGE7+GUB4-5+MAGEA1+CAGE+CYFRA21-1+NSE+SCCA	37.76/-/0.660	(138)
	P53+PGP9.5+SOX2+GAGE7+GUB4-5+MAGEA1+CAGE+CYFRA21-1+CEA	52.26/77.46/-	(139)
Liver tumor	PCNA+AAGAB+CDC37L1+TAF7L+DUSP6+ZIC2+AFP	90.9/-/0.889	(140)
	DNMT3A+p16+HSPA5+HSP60	75/85/-	(141)
	PAX5+PTCH1+GNA11	68.0/94.1/-	(142)
Colorectal tumor	CKS1B+S100A11+maspin+ANXA3+eEF2	51.11/-/0.75	(143)
Ovarian tumor	CFL1+EZR+CYPA	55.56/81.31/0.762	(144)
	LRDD+FOXA1	58.1/87.5/-	(145)
	TP53+GNAS+NPM1	51.2/86.0/-	(2)
	BARD1+CA125	91/95/-	(146)
Prostate tumor	IFIT5+PAH+FCER2+CPOX	65.0/83.3/0.784	(39)
Breast tumor	CEBPA+CEP55+GATA3+HRAS+PTCH1+RalA	78.9/90.2/0.916	(60)
	KJ901215+FAM49B+HYI +GARS+CRLF3	38.78/85.94/-	(147)
	p53+RalA+p90+NYESO -1+HSP70	38/90/-	(148)
	HSPA4+ENO1+PRDX6+PRPF19+MMP14	-/-/0.97	(149)
Esophageal tumor	MAGEA1-IgG+MAGEA4-IgG+MAGEA6-IgG+MAGEA12-IgG	51.8/84.5/0.698	(150)
	CAGE+CTAG2+MAGE-A4+Trim21+RalA	83.08/72.73/0.880	(151)
	NSG1+CEA+CYFRA21-1+SCC	67.3/88.3/0.758	(152)
	ALDOA+ENO1+p53+NY- ESO -1	68.8/90.4/0.860	(54)
	CAST+HDAC1+HSF1+PTMS+ZPR1	62.50/90.08/0.83	(153)
	CETN2+POFUT1	85.09/56.25/0.781	(154)
	c-myc+p62+RalA+p53+Sui1+NY- ESO -1	72/76/-	(155)
	P53+PGP9.5+SOX2+GAGE7+GBU4-5+MAGE A1+CAGE)	49.58/92.98/-	(156)
Gastric tumor	NRAS+MFGE8+PTP4A1+RRAS2	70.8/85.9/0.87	(36)
	CLDN18+CAGE1+CTAG1A+PBRM1+RASSF7+IMMP2L+COPB1	-/-/0.885	(157)
	RAE1+PGK1+NPM1+ARF4	75.6/87.7/0.857	(158)
Pancreatic tumor	CEACAM1+DPPA2+DPPA3+MAGEA4+SRC+ TPBG+XAGE3	82.8/68.4/0.85	(159)
	CLDN17+CA19.9	85/86/0.95	(160)

SN, Sensitivity; SP, Specificity; AUC, Area Under the Curve.

## 5 Development prospects and challenges for TAAbs

TAAbs exhibit unique advantages in the early diagnosis, dynamic monitoring, and prognostic assessment of tumors, such as high specificity, long - term stability, and early detection. However, their clinical translation is restricted by issues like limited sensitivity, insufficient tumor - specificity, substantial influence of individual heterogeneity, and difficulties in real - time efficacy assessment and recurrence monitoring. On the other hand, due to differences in detection methods, sample sizes, selection of study endpoints, and patient and tumor heterogeneity, current research on TAAbs varies significantly. There is a lack of standardized interpretation and prospective validation studies in large, diverse populations. Based on a systematic analysis of the biological characteristics of TAAbs, detection technologies, and the current state of clinical research, we propose the following three most promising and feasible future research directions:

### 5.1 Constructing a multi - omics integrated TAAbs profile to enable precise tumor subtyping and early warning

Across various tumor types, the positivity rate of TAAbs generally ranges from 10% to 30% (50). Multiple studies have demonstrated that the combined testing of autoantibodies (including combinations of autoantibodies or co - testing with other tumor markers) can significantly enhance sensitivity. Moreover, many TAAbs are not specific to a particular tumor. TAAbs positivity only indicates the presence of a tumor; it cannot precisely localize or classify the tumor, which needs to be confirmed through imaging studies, pathological biopsies, and other methods. Inspired by the concept that autoimmune diseases have characteristic autoantibody profiles, different tumor types, and even different molecular subtypes of the same tumor, should display specific TAAbs combination profiles. Future research could integrate high - throughput protein arrays, phage display technology, and clinical multi - omics data (genomic, transcriptomic, and proteomic) to construct a “super - profile” of TAAbs covering various tumors. By screening large sample cohorts (including prospective screening cohorts), high - sensitivity and high - specificity TAAbs combinations could be identified for early warning, tumor localization, and molecular subtyping. The aim is to overcome the current limitation of TAAbs - based methods, which is their inability to achieve precise localization, and to develop a minimally invasive, low - cost, and high - coverage early screening tool based on peripheral blood. This tool would be particularly suitable for regular screening of asymptomatic high - risk populations.

### 5.2 Analyzing the dynamic evolution of TAAbs subtypes to enhance the value of treatment monitoring and immunological intervention

Current studies on autoantibody subtypes primarily focus on IgG.However, some literature indicates that IgA is the most common subtype in early - stage ovarian tumor, while IgG and IgM are more frequently observed in advanced stages (161). Different antibody subtypes exhibit significant differences in mucosal immunity, complement activation, ADCC effects, and half - life, which may reflect shifts in the type of immune response during tumor progression. Furthermore, given the relatively long half - life of TAAbs (7–30 days), they are not sensitive enough to reflect short - term changes in tumor burden (162–165), whereas subtype shifts may provide more dynamic information. Therefore, future efforts could conduct prospective longitudinal studies to systematically analyze the subtype distribution of TAAbs (IgA, IgG1–4, IgM, IgE, etc.) across different tumor types and stages, as well as their correlation with tumor burden and treatment response.This would enable the establishment of dynamic monitoring models based on subtype proportions or conversion timelines for real - time efficacy assessment and recurrence prediction.Concurrently, immunological intervention strategies to induce or block specific TAAbs subtypes, such as vaccines or antibody therapies, could be explored, providing biomarkers and targets for the development of novel immunotherapies targeting B cells or plasma cells.

### 5.3 Exploring the functions of TAAbs - antigen immune complexes and their interactions with the tumor microenvironment to reveal their dual roles in disease progression and protection

Current research has mainly focused on free TAAbs. However, immune complexes formed between tumor antigens and autoantibodies can influence tumor - associated macrophages, dendritic cells, and the complement system via Fc receptor - mediated signaling pathways, thus exerting dual pro - tumor and anti - tumor effects.By investigating TAAbs - antigen immune complexes and clarifying their specific impacts on antigen presentation, immune cell polarization, and tumor angiogenesis, new therapeutic targets may be identified.Simultaneously, TAAbs - antigen immune complexes integrate dual information from autoantibodies and antigens, potentially reflecting both the immune response and tumor burden, thereby overcoming the limitations of TAAbs in real - time monitoring (166–168).

In conclusion, the detection of TAAbs presents potential as a supplementary instrument for tumor diagnosis and treatment. Nonetheless, considerable obstacles persist in its clinical implementation, necessitating additional investigation.
